# The effect of transcranial direct current stimulation (tDCS) on cognitive function recovery in patients with depression following electroconvulsive therapy (ECT): protocol for a randomized controlled trial

**DOI:** 10.1186/s12888-024-05567-9

**Published:** 2024-02-16

**Authors:** Renqin Hu, Junyao Li, Yulin Lu, Huirong Luo, Yinlin Zhang, Xueqian Wang, Zheng Zhang, Qinghua Luo

**Affiliations:** 1 Department of Psychiatry, the First Affiliated Hospital of Chongqing Medical University, Chongqing, 400016 China; 2 Department of Psychiatry, People’s Hospital of Chongqing Banan District, Chongqing, China

**Keywords:** Electroconvulsive therapy (ECT), Transcranial direct current stimulation (tDCS), Depressive Disorder, Dorsolateral prefrontal cortex (DLPFC), Cognitive side effects, Cambridge Neuropsychological Test Automated Battery (CANTAB)

## Abstract

**Background:**

Electroconvulsive therapy (ECT) is a highly effective treatment for depressive disorder. However, the use of ECT is limited by its cognitive side effects (CSEs), and no specific intervention has been developed to address this problem. As transcranial direct current stimulation (tDCS) is a safe and useful tool for improving cognitive function, the main objective of this study was to explore the ability to use tDCS after ECT to ameliorate the cognitive side effects.

**Methods:**

60 eligible participants will be recruited within two days after completing ECT course and randomly assigned to receive either active or sham stimulation in a blinded, parallel-design trial and continue their usual pharmacotherapy. The tDCS protocol consists of 30-min sessions at 2 mA, 5 times per week for 2 consecutive weeks, applied through 15-cm^2^ electrodes. An anode will be placed over the left dorsolateral prefrontal cortex (DLPFC), and a cathode will be placed over the right supraorbital cortex. Cognitive function and depressive symptoms will be assessed before the first stimulation (T0), after the final stimulation (T1), 2 weeks after the final stimulation (T2), and 4 weeks after the final stimulation (T3) using the Cambridge Neuropsychological Test Automated Battery (CANTAB).

**Discussion:**

We describe a novel clinical trial to explore whether the administration of tDCS after completing ECT course can accelerates recovery from the CSEs. We hypothesized that the active group would recover faster from the CSEs and be superior to the sham group. If our hypothesis is supported, the use of tDCS could benefit eligible patients who are reluctant to receive ECT and reduce the risk of self-inflicted or suicide due to delays in treatment.

**Trial registration details:**

The trial protocol is registered with https://www.chictr.org.cn/ under protocol registration number ChiCTR2300071147 (date of registration: 05.06.2023). Recruitment will start in November 2023.

**Supplementary Information:**

The online version contains supplementary material available at 10.1186/s12888-024-05567-9.

## Introduction

### Background

Electroconvulsive therapy (ECT) stands out as an effective and rapid treatment for major depressive disorder (MDD) patients [1]. However, concerns about potential cognitive side effects (CSEs) deter patients from consenting to ECT [2]. While most deficits, including memory, executive function, attention, and processing speed, are transient [3, 4], sensitive assessment tools suggest that spatial recognition memory impairment can persist for more than a month following ECT [5, 6]. Furthermore, patients reported a deleterious effect on memory persisting 24 weeks post-ECT using Global Self-Evaluation—Memory [7, 8].

The underlying cause of cognitive impairment following ECT remains unclear. Hippocampal enlargement after ECT is posited to have a significant correlation with cognitive dysfunction [9–12], potentially attributable to the interference of neuroplastic alterations with existing synaptic connections following ECT [9, 10]. Besides, a consistently replicated finding points to reduced prefrontal cerebral blood flow (CBF) and metabolism post-ECT [13–16]. Moreover, challenging conventional perspectives, the question arises: are the antidepressant effects and the CSEs of ECT a consequence of the seizure, the electrical stimulation, or a combination of both [17–19]?

To mitigate CSEs, researchers have pursued various approaches. Significantly, advancements in electrode placement and electrical parameters, such as high dosage right unilateral ECT and ultra-brief pulse width ECT, have effectively reduced CSEs [20–22]. However, some studies indicate that compared to right unilateral ECT, bilateral ECT demonstrates higher efficacy and may offer a quicker onset of action [23–25]. Similarly, brief pulse width right unilateral ECT showed slightly higher effectiveness in treating depression with fewer sessions than ultra-brief pulse width right unilateral ECT [20]. In addition, extensive research has been dedicated to pharmacological interventions [26]. Notably, although promising medications like liothyronine [27], memantine [28, 29], and galantamine [30] have been reported to potentially alleviate CSEs, further rigorous trials are indispensable to verify their appropriateness for routine clinical application. In sum, exploring alternative strategies becomes imperative.

Transcranial direct current stimulation (tDCS) is a non-invasive, effective, and affordable brain stimulation tool with mild side effects [31, 32]. Consequently, tDCS has been widely applied in the field of neuropsychiatry to enhancing cognitive function. Studies have demonstrated that tDCS applied to frontal and temporal regions, with the most commonly stimulated site being the dorsolateral prefrontal cortex (DLPFC), can modulate specific neural circuits, thereby elicit a transdiagnostic enhancement of working memory and attention [33]. This effect has been observed in various conditions, including Depressive Disorder [34], Schizophrenia [35], Bipolar Disorder [36], Attention-Deficit/Hyperactivity Disorder [37], Alzheimer's disease [38] and Parkinson's disease [39]. Furthermore, some articles have revealed that anodal tDCS over the DLPFC can decrease response time [40, 41], improves executive function [42], boosts episodic memories [43, 44] and promotes other cognitive performance [45] in healthy participants. Additionally, the increased CBF [46–48] and cortical activity [49–51] induced by anodal stimulation are likely to improve the ECT-induced reduction in frontal CBF and metabolism [13, 14, 16]. However, such research remains largely unexplored to date. Therefore, utilization tDCS to ameliorate CSEs in ECT appears promising and merits further investigation.

## Method

### Objective

The primary objective of this study is to determine whether the implementation of active tDCS after completing the ECT course accelerates recovery from the CSEs, especially the spatial recognition memory.

Secondary, the study aims at examining the long-term (after 4 weeks) effects in improving cognitive function and the whether the implementation of tDCS after the ECT course decrease the relapse of depressive symptoms.

### Study design and setting

**Fig. 1:**
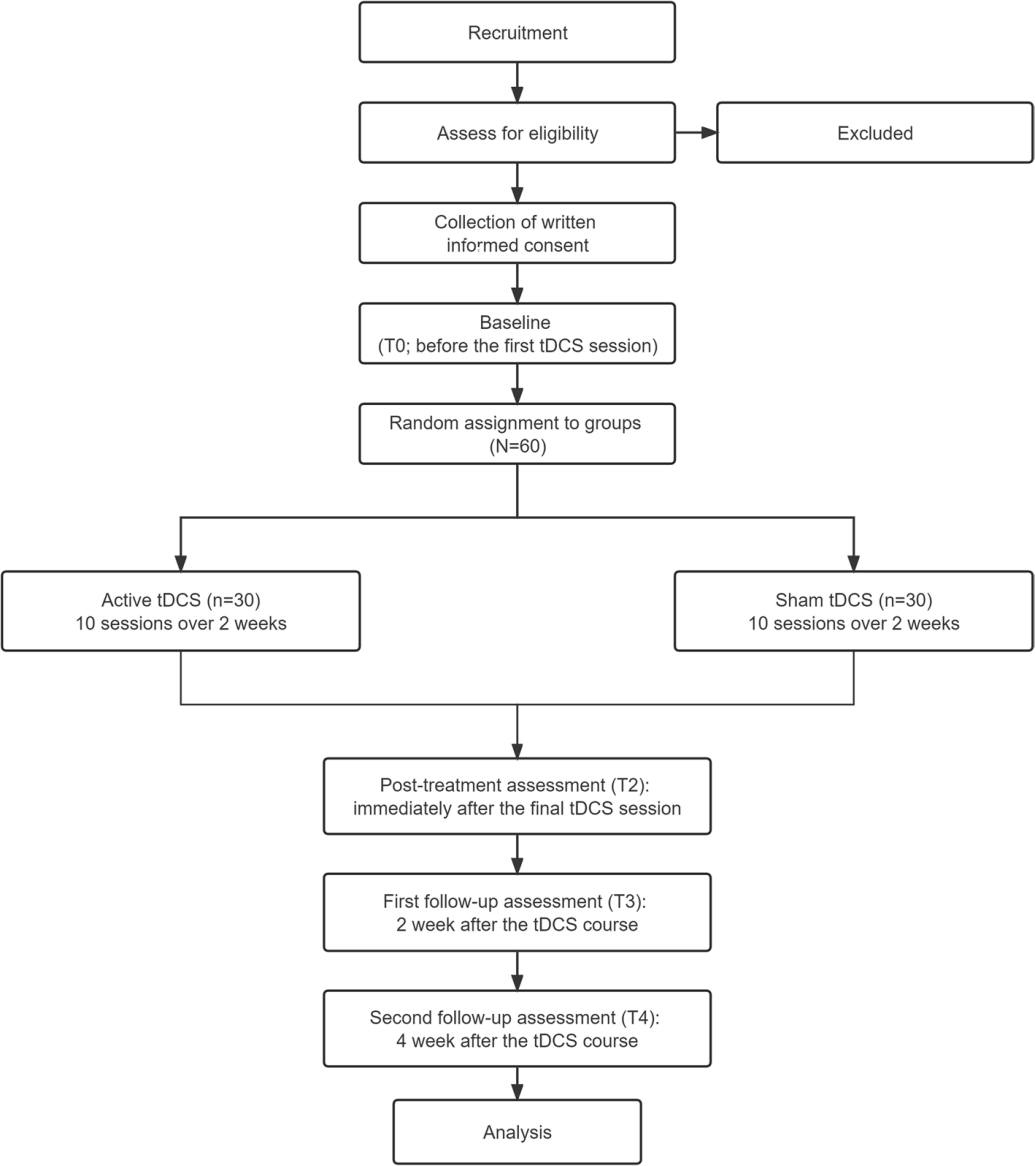
Flowchart of the study design

This is a randomized, double-blind, sham-controlled, interventional trial. 60 eligible participants will be recruited within two days after the last ECT session and randomly allocated into two groups in a 1:1 ratio: active tDCS (intervention group) and sham tDCS (control group). Assessments will be conducted at the following time points: before the first tDCS session (T0), immediately after the last tDCS session (T1), 2 weeks post-tDCS (T2), and 4 weeks post-tDCS (T3), using the Cambridge Neuropsychological Test Automated Battery (CANTAB). Table 2 shows schedule of enrolment, intervention, and assessments. Throughout the study, there was no interference or restriction for the patient ‘s physician in charge to adjust psychiatric medications according to the patient’ s condition. Figure 1 represents the research procedure schematically.

This study, approved by the Research Ethics Committee of the First Affiliated Hospital of Chongqing Medical University (Approval Number: 2023–203) and registered with the China Clinical Trials Center (Registration Number: ChiCTR2300071147), will be conducted at one of Southwest China's largest hospitals, the First Affiliated Hospital of Chongqing Medical University, where approximately 40 to 60 patients receive ECT daily. The study design is in accordance with the 2013 Standard Protocol Items: Recommendations for Interventional Trials (SPIRIT) statement [52].

### Recruitment

Participants will be recruited from the Psychiatry Department by the researchers. The study will be advertised on the hospital's website, and we will distribute leaflets to the patients two days prior to their discharge. Prior to screening, all potential participants will receive verbal and written explanations about the study's procedures and potential benefits or risks. Every participant will voluntarily sign the informed consent form (Supplementary Material: informed consent) and can withdraw from the trial at any time. Written informed consent will be obtained from all participants and their legal guardians before their formal inclusion in the study. Compliance will be improved by using WeChat (a popular social communication app in China) for appointment reminders and offering transportation cost reimbursements to reduce dropout among financially constrained participants.

### Participants

Participants will be assessed based on inclusion and exclusion criteria highlighted in Table 1. Throughout the study, researchers may opt to discontinue a participant's involvement due to urgent medical circumstances or if the participant's condition changes to the extent that they no longer meet the eligibility criteria for participation.

**Table 1:** Inclusion and exclusion criteria

**Inclusion criteria:**
1. Aged 18 to 65 years
2. Right-handed
3. Diagnosed with MDD based on DSM-5 criteria by at least one qualified psychiatrist
4. 24-item Hamilton Rating Scale for Depression (HAMD-24) score ≥ 17
5. Completed ECT treatment
6. Capable of comprehending and completing the study assessments
7. Written informed consent provided by patients and/or their legal guardians
**Exclusion criteria:**
1. Severe systemic illnesses (e.g., hepatic, renal, respiratory, cardiovascular, endocrine, hematological, oncological diseases)
2. Diagnosis of schizophrenia, bipolar disorder, dementia, substance dependence or abuse, or any primary psychotic disorder according to DSM-V criteria
3. Skin lesions or dermatological issues at electrode sites
4. Metal implants or electrically sensitive devices
5. Neurological conditions (e.g., stroke, seizure disorders) affecting cognition or treatment response
6. ECT within the past six months
7. Known hypersensitivity or contraindication to concurrent ECT medications
8. Pregnant or lactating
9. Receiving transcranial magnetic stimulation or other neuromodulation treatments
10. Participation in another concurrent clinical trial

### Randomization

Random sequences will be generated by a statistician with no other connection to the trial using Microsoft Office Excel 2017. The randomization sequence list will remain concealed from the investigators. Allocation concealment will be achieved through consecutively numbered, opaque, and sealed envelopes. A research nurse, uninvolved in any other study procedures, will assign the participants to their group to ensure objectivity and minimize bias.

### Blinding and unblinding

The tDCS treatment device consists of two parts: a computer and a small stimulator. To prevent participants from being directly aware of their group assignment, the treatment is carried out in a dedicated room, where the patient will sit on a couch positioned away from the computer and the stimulator will be placed in an opaque bag behind the patient. The psychiatrist administering tDCS knows the group assignments but cannot share this with participants or engage in the study’s evaluation or analysis. Additionally, the assessors and data analysts will be kept unaware of the intervention groups. To evaluate the adequacy of blinding procedures, participants will be invited to make an educated guess regarding their treatment assignment after the tDCS sessions.

If there is a suspicion of a severe adverse reaction or if clinical psychiatrists determine it is essential for participant safety, disclosure of group membership may be required.

### Intervention

tDCS will be administered using a battery-driven direct current stimulator (EM8010S, Yimai Medical Technology Co., Ltd. Wuhan, China) via a pair of identical squares (3 × 5 cm) saline-soaked sponge electrodes secured in place with elastic bands. The anodal electrodes will be positioned over the F3 site, corresponding to the DLPFC following the EEG10– 20 international system. Conversely, the cathodal electrode will be placed above the Fp2 site, corresponding to the right supraorbital region. Within two days after completing the ECT course, all study participants will receive tDCS five times per week, once daily, for two consecutive weeks. In the active tDCS condition, each session will deliver a direct current of 2 mA for 30 min. In the sham tDCS condition, stimulation will be administered using the same active tDCS montage. The stimulation intensity will be set at 2 mA, but the current will be applied for 1 min with a 30-s ramp-up and ramp-down. In prior studies [53], this approach ensured participant blinding regarding the stimulation type (active vs. sham).

During the intervention, participants will continue their usual pharmacotherapy, but transcranial magnetic stimulation or other neuromodulation treatments are forbidden. The intervention will be discontinued if there are any severe adverse effects or withdrawal. This trial is deemed of minimal risk to study participants. Therefore, there are no provisions for ancillary or posttrial care.

### ECT regimen

The ECT regimen, initially up to four times in the first week and then adjusted to three times weekly, is determined by the treating psychiatrist. It will be conducted using a Thymatron DGx apparatus, with bitemporal electrode placement 5 cm above the outer angle of the orbit. The initial electric charge is calculated using the half-age method, with subsequent dosages adjusted based on EEG seizure activity, increasing by 5% each session. The ECT uses square-wave stimulation at 900 mA, with 125 bidirectional pulses per second and a brief-pulse width of 1.5 ms. Anesthesia involves intravenous atropine (0.5 mg), propofol (2 mg/kg), and succinylcholine (1 mg/kg). Subsequent dosages will be adjusted according to the patient's response to achieve appropriate seizure morphology. We will assess seizure adequacy based on morphology and duration, including polyspike wave, 3-Hz spike and wave activity, and postictal suppression [54]. ECT effectiveness will be defined by induced electroencephalograph seizure durations exceeding 25 s [55].

### Outcome

To assess cognitive changes, we will employ the CANTAB, a tool previously demonstrated to be highly sensitive in evaluating the cognitive side effects of ECT [5, 6]. Additionally, we have chosen a set of five tests designed to assess frontal lobe function. To mitigate potential learning effects, different parallel versions of the same test will be employed during various assessment phases. Assessments of cognitive function and depressive symptoms will be conducted before the first tDCS session (T0), immediately after the last tDCS session (T1), 2 weeks post-tDCS (T2), and 4 weeks post-tDCS (T3). The detailed visit procedure is shown in Table 2.

**Table 2:** Schedule of enrolment, intervention, and assessments

	Enrolment	Baseline	Intervention	Follow-up	
		Before tDCS	After tDCS	after 2 weeks	After 4 weeks
Time point		T0	T1	T2	T3
Enrolment					
Eligibility screening	√				
Informed consent	√				
Allocation	√				
Demographic data		√			
Intervention					
Active tDCS			√		
Sham tDCS			√		
Assessments					
CANTAB					
SRM		√	√	√	√
SWM		√	√	√	√
RVP		√	√	√	√
VRM		√	√	√	√
SOC		√	√	√	√
HAMD-24		√	√	√	√
SDS		√	√	√	√
tDCS-AEQ^a^			√		
Blinding			√		

*a: test after every tDCS session*

*CANTAB: Cambridge Neuropsychological Test Automated Battery, HAMD-24: Hamilton depression rating scale; RVP: Rapid visual information processing; SDS: Self-rating depression scale; SOC: Stockings of Cambridge; SRM: Spatial recognition memory; SWM: Spatial working memory; VRM: Verbal Recognition Memory; tDCS, transcranial direct current stimulation; tDCS-AEQ: Transcranial direct current stimulation adverse effects questionnaire*

### Primary outcome

The primary outcome measured is the change in Spatial Recognition Memory (SRM), which assesses spatial recognition memory using a forced-choice paradigm. In the first phase, participants are presented with a series of 5 white boxes, each at a different spatial location on the screen. During this phase, participants are instructed to remember the locations of these white boxes. In the second phase, two boxes are simultaneously presented. The target box occupies a location from the first phase, while the distractor box is placed in a previously unused location. Participants must recognize and click on the target box. This process is repeated four times, with new target and distractor locations in each test. The percentage of correct responses will be recorded as part of this assessment.

### Second outcome

1. Spatial Working Memory (SWM) assesses the subject's ability to retain and manipulate spatial information in working memory. The outcome measures include between errors, within errors, and strategy employed. A high strategy score represents poor use of strategy, and a low score equates to effective use.
2. Verbal Recognition Memory (VRM) evaluates immediate and delayed verbal information memory. Outcome measures include the total number of correct words in the free recall phase, the total of all correctly identified words in the immediate recognition phase, and the delayed recognition phase.
3. Rapid Visual Information Processing (RVP) assesses visual sustained attention. Outcome measures include mean latency and total correct responses.
4. Stockings of Cambridge (SOC) evaluates spatial planning and spatial working memory. Outcome measures include the number of n-move problems solved in the minimum moves, mean moves for n-move problems, mean initial thinking time for n-move problems, and mean subsequent thinking time for n-move problems.
5. Assessing the degree of depressive symptom with the Hamilton Depression Rating Scale (HAMD-24) [56] and the Self-rated Depression Scale (SDS) [57, 58].

### Harms

All participants will complete the tDCS adverse effects questionnaire (tDCS-AEQ) after each tDCS session to evaluate potential adverse effects of the intervention. Common adverse reactions, as indicated by a systematic review [59], include tingling sensations, itching, mild skin redness, and discomfort in the stimulation area. Participants will rate their experienced adverse events on a 0 to 5 scale. Serious adverse events (SAEs) or reactions are considered unlikely based on the review. Only unexpected serious adverse events or reactions unrelated to these clinical procedures will be reported as SAEs.

### Date collection methods and management

CANTAB is a computer-administered battery of tasks, and the primary outcome variables from the CANTAB tasks will be automatically calculated by the software. A clinical senior psychiatrist blinded to the group allocation will assess the severity of depressive symptoms using HAMD-24.

All participant demographic and scale related results will be recorded in the case report form (CRF). For patient privacy, irrelevant personal information like names, contact numbers, and addresses will be omitted during data entry, using patient numbers for identification. The original CRFs are securely stored and accessible only to the project leader and principal investigator.

Our trial does not have a data monitoring committee, but the subject group has a dedicated person to oversee. We will notify patients to come for tDCS via WeChat (a popular social communication app in China) and reimburse their transportation costs to improve patient compliance.

## Statistical analysis

### Sample size

Based on primary cognitive function outcome—the SRM correct rate [5], we calculated the sample size. Sample size was determined via PASS software (PASS 15, NCSS, LLC. Kaysville, UT, USA) for a power of 0.80 and a two-tailed α level of 0.05. Parameters were derived from a prior study [5] (mean SRM correct rate at 1-month follow-up: 64.67%, SE: 3.88, SD: 15.03, dropout rate: 37.5%). Calculations yielded permissible error (δ) = 16.20 (20% of mean), and σ = 15.03. Recruitment won't exceed original participants; thus, 24 patients per group are needed. Thus, 24 patients per group are needed. Based on a 20% dropout rate, each group ended up with 30 participants.

### Statistical methods

To assess the normality or approximate normality of the data, we will employ a combination of histograms and the Shapiro–Wilk test. For normally or approximately normally distributed data, we will report the results as mean ± standard deviation (SD). If the data exhibit skewness, we will express the results as medians and quartiles. Categorical data will be presented as absolute numbers and percentages. Appropriate statistical tests, such as t-tests, nonparametric tests, or chi-square tests, were used in assessing differences in baseline characteristics between groups.

Consideration of repeated measurement data, we employed three strategies for presentation. Firstly, a line graph was utilized to depict values (whether rates or averages) at distinct time points. Secondly, we applied mixed regression models to assess both between-group and time effects for both primary and secondary outcomes. We will employ the stepwise selection approach to construct parsimonious regression models. These adjustments will account for additional factors that may potentially influence these associations, including age, ECT sessions, HAMD-24 score, SDS score, gender, current medication usage, disease duration, etc. Age and gender were entered in all models regardless of their statistical significance. Thirdly, we examined simple effects at different time points and adjusted *p*-values to reduce the risk of false positives.

No interim analysis and subgroup analysis will be performed. Transforming the data into a longitudinal format to meet the fitting requirements of mixed regression models. The statistical analysis of our data will be conducted using SPSS version 25.0 and R version 4.1.3. We define statistical significance as a two-tailed *p*-value of less than 0.05.

### Protocol amendments

Significant protocol changes will be informed to the Ethics Committee. If approved, updates will be notified in writing to involved parties, and the Chinese Clinical Trial Registry record will be adjusted.

### Dissemination policy

Before the first patient enrolls, trial details will be available on the Chinese Clinical Trial Registry. All kinds of results will be shared. They will be published and discussed in international conferences and peer-reviewed journals.

## Discussion

This is the first RCT aimed at using tDCS to assist in the recovery of CSEs caused by ECT in depression patients. Given that CSEs might hinder patients from opting for ECT [60], identifying a rehabilitation strategy could assist patients in alleviating these concerns.

Depressive patients exhibit an imbalance in cortical metabolism between the two hemispheres, with lower metabolism observed in the left frontal lobe compared to the right frontal lobe [61, 62]. ECT can induce a short-term reduction of CBF and metabolism in frontal lobe, but this alteration subsequently normalizes within around one month [13].

By utilizing the anode at left DLPFC and the cathode at right DLPFC, tDCS aims to rectify the cortical imbalance in depression, ultimately ameliorating depressive symptoms [61]. Although the mechanisms underlying the impact of tDCS on cognitive function remain not entirely clear, early research suggests that tDCS may elicit long-term effects extending beyond the stimulation period [49, 63, 64]. Otherwise, these enduring effects are likely mediated through the modulation of N-methyl-D-aspartate and gamma-aminobutyric acid receptor activity [65, 66].

Cognitively, depression in the elderly is intricately linked with dementia; past studies have suggested that depressive symptoms might be a precursor to dementia [67, 68]. Despite improvements in depressive symptoms, elderly patients often continue to exhibit deficits in visuospatial ability, information-processing speed, and delayed memory [69]. This complexity adds to the challenge of distinguishing whether cognitive deficits in elderly patients post-ECT are due to ECT itself or pre-existing conditions. Further research indicates that while ECT treatment may lead to short-term cognitive decline in elderly patients [70], there is potential for long-term cognitive improvement [71]. Therefore, enhancing cognitive function during symptom remission in elderly patients with depression is of critical clinical importance, a goal that requires further dedicated efforts [72].

There are also limitations to this RCT. Firstly, our study did not include patients over the age of 65, a demographic with a significant need for cognitive improvement. Secondly, The lack of pre-ECT cognitive assessments in our study restricted our thorough evaluation of cognitive functions in patients with depression. Thirdly, due to financial constraints as an investigator-initiated study, there is no independent Trial Steering or Monitoring Committee. However, a research management group composed of study researchers will oversee daily operations and data authenticity. Fourthly, administering tDCS in a hospital poses challenges for discharged patients in terms of transportation, potentially leading to higher drop-out rates. lastly, the research is conducted at a single site, potentially limiting the external validity of the findings.

### Supplementary Information

**Additional file 1. **Informed Consent Form.

## Data Availability

Not applicable.

## Abbreviations

CANTAB: Cambridge Neuropsychological Test Automated Battery
CBF: Cerebral blood flow
DLPFC: Dorsolateral prefrontal cortex
DSM-5: Diagnostic and Statistical Manual of Mental Disorders, Fifth Edition
ECT: Electroconvulsive therapy
HAMD-24: 24-Item Hamilton Rating Scale for Depression
MDD: Major depressive disorder
RCT: Randomized controlled trial
RVP: Rapid visual information processing
SDS: Self-rated depression scale
SRM: Spatial recognition memory
SWM: Spatial working memory
SD: Standard deviation
tDCS: Transcranial direct current stimulation
tDCS-AEQ: Transcranial direct current stimulation adverse effects questionnaire
