# Supplementary material for: The effect of transcranial direct current stimulation (tDCS) on cognitive function recovery in patients with depression following electroconvulsive therapy (ECT): protocol for a randomized controlled trial
Source: BMC Psychiatry. 2024 Feb 16;24:130. doi: 10.1186/s12888-024-05567-9 (PMC10870564; doi:10.1186/s12888-024-05567-9)
Supplement: Supplementary file 1 — Additional file 1. Informed Consent Form. [file 12888_2024_5567_MOESM1_ESM.docx]

Informed Consent Form

###### Introduce

Depressive Disorder is an affective disorder manifested by either a dysphoric mood or loss of interest or pleasure in usual activities, and the mood disturbance is prominent and relatively persistent. Electroconvulsive therapy (ECT) is an effective, rapid and safe treatment for patients suffering from Depressive Disorder. However, the main cognitive domain affected by ECT such as anterograde and retrograde memory, attention, executive function, and processing speed, have not been well addressed. As you recently completed the ECT course, we sincerely introduce this study called to you.

###### The name of the study and approval for its implementation

The title of this study: “The effect of transcranial direct current stimulation (tDCS) on cognitive function recovery in patients with depression following electroconvulsive therapy (ECT): protocol for a randomized controlled trial”.

This study was approved by the Research Ethics Committee of the First Affiliated Hospital of Chongqing Medical University (Approval Number: 2023-203) and registered in the Chinese Clinical Trial Registry (identifier: ChiCTR2300071147).

###### Objective

In this study, we will measure and evaluate cognitive function using Cambridge Neuropsychological Test Automated Battery, after completing ten sessions of transcranial direct current electrical stimulation to determine the effectiveness of the study. If it becomes clear that the cognitive side effects were improved, this will lead to the development of new methods to help numerous patients who are reluctant to undergo ECT but need ECT.

###### Transcranial direct current electrical stimulation (tDCS)

tDCS is a non-invasive, effective and affordable technique of brain electric stimulation therapy which uses constant, low current (2 mA in this study) delivered via electrodes placed on various locations on the scalp, which have various effects such as improving mental symptoms and cognitive function.

###### Inclusion and exclusion criteria

- 1. **who will be included?**

1. 18 to 65 years of age;

2. Right-handed;

3. Diagnosis of MDD according to the Diagnostic and Statistical Manual of Mental Disorders, fifth edition (DSM-5), criteria according to at least one professional psychiatrist;

4. Completed ECT course;

5. Able to understand and complete the assessments included in the study;

6. Provision of written informed consent by patients and/or their guardians.

**5.2 who will be excluded?**

1. The presence of severe systemic illnesses including but not limited to severe hepatic, renal, respiratory, cardiovascular, endocrine, hematological or oncological diseases;

2. Diagnosis with schizophrenia or any other primary psychotic disorder, bipolar disorder, dementia, or substance dependence or substance abuse according to DSM-V criteria;

3. Skin lesions or dermatological disorder at the site of electrodes;

4. Metal implants or any electrically sensitive support devices;

5. Neurological conditions (e.g., stroke, seizure disorders) that can affect cognition or response to treatment;

6. ECT within the past six months before admission;

7. Known hypersensitivity or contraindication to concomitant medications used for ECT;

8. Pregnant or lactating;

9. Undergoing transcranial magnetic stimulation (TMS) or other neuromodulation treatments;

10. Participation in a concurrent clinical trial.

###### tDCS plan

You will receive 5 tDCS sessions per week once daily for 2 consecutive weeks within 2 days after completing ECT course and continue your usual pharmacotherapy. An anode will be placed over the left dorsolateral prefrontal cortex (DLPFC), and a cathode will be placed over the right supraorbital cortex. For the objective of the active vs. sham tDCS study, you will be randomly assigned to either the active or sham group. In the sham group, the current will only be applied for 30 s ramped up and 30 s ramped down. The total duration of this study is 6 weeks.

The following is how the study will be administered.

1. Firstly, you will be screened to determine whether you meet the entry criteria and do not meet the exclusion criteria.
2. Upon confirmation of participation, you will be randomly assigned to either the active or sham group. The evaluator conducting the psychological assessment and you were not informed which group you were until the completion of the study. Before the first stimulation, we will collect your general demographic data and date of cognitive function assessments.
3. The tDCS session will be performed following the plan. During the study, you need to report adverse effects.
4. After completing the tDCS course, we will evaluate your cognitive function again.

Please note the discontinuation rules for participants include withdrawal of consent for any reason, development of a coincidental health problem, increased incidence of self-harm or self-injury, or other diseases for which treatment may affect the assessment of tDCS.**Figure 1**. Flowchart of the study design. tDCS, transcranial direct current stimulation


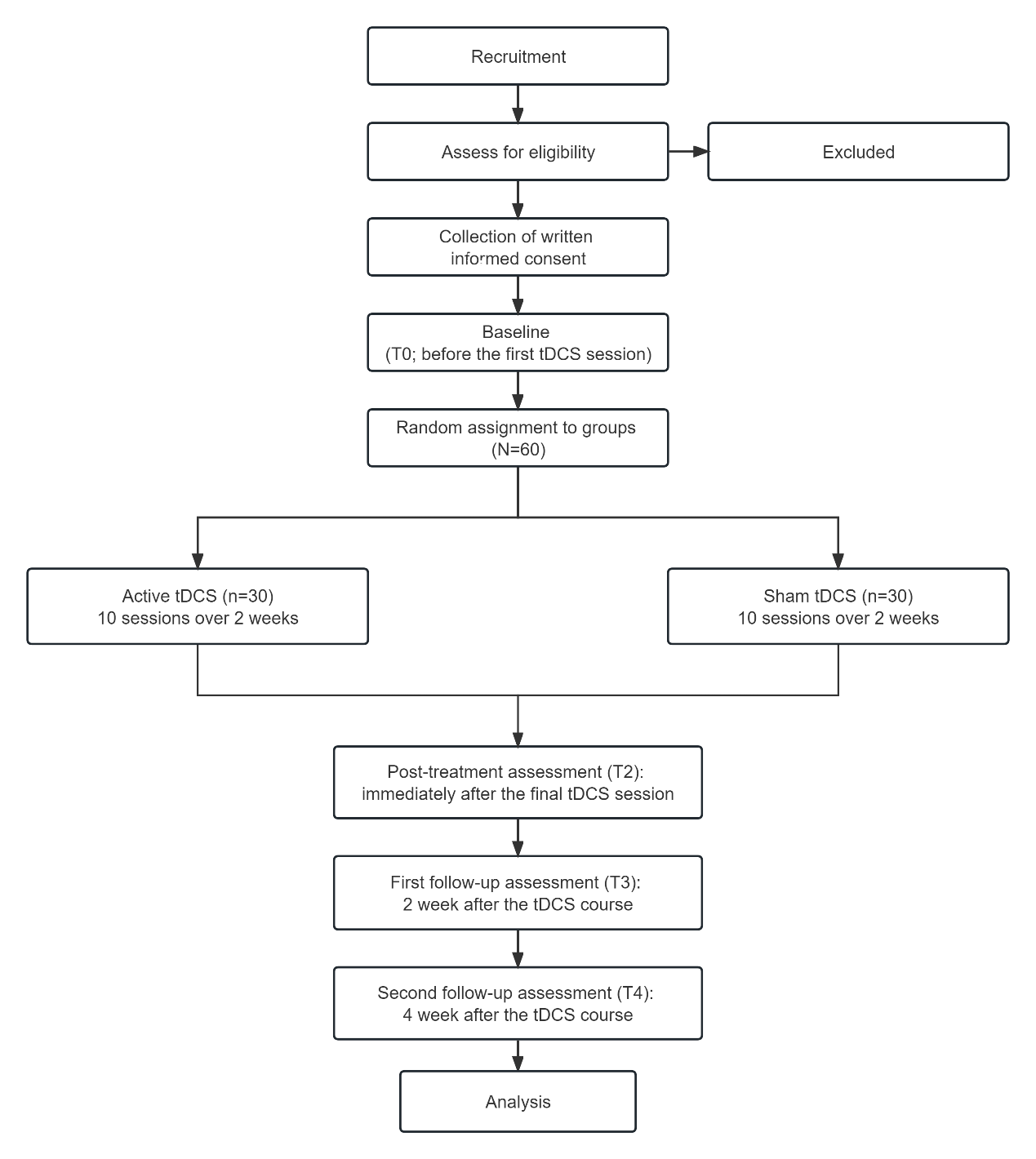


###### Cognitive function assessment

Cognitive function will be assessed before the first stimulation (T0), immediately after the final stimulation(T1), after 2 weeks (T2), and after 4 weeks (T3) using the Cambridge Neuropsychological Test Automated Battery (CANTAB). All tests take about 40 minutes. The CANTAB is an operational and widely used tool for assessing cognitive function, and the evaluator will teach you how to do it. Here is a description of each test:

Spatial Recognition Memory (SRM): In the first phase, a series of 5 white boxes are individually presented on the screen, each at a different spatial location. In this phase, participants will be instructed to remember the location of white boxes. In the second phase, two boxes are presented simultaneously. The target occupies a location used during the first phase. The distractor is present in a previously unused location. During the second phase, the targets are presented in the reverse order in which they were presented in the first phase. The participants must recognize and click on the target. This test will be repeated 4 times, using new target and distractor locations on each test.

Spatial Working Memory (SWM): Several coloured boxes are presented on the screen, and the participants are instructed to click on boxes to reveal a blue token hidden below. They can then use this token to fill an empty column on the right side of the screen. The tokens are not hidden beneath the same box within trials, and the colour and position of the box constantly change after the token is moved to the column. There are four test trials with two, three, four, six and eight boxes.

Verbal Recognition Memory (VRM): A series of 18 words or phrases are individually shown to participants on the screen, and the participants are instructed to remember the target words. In the Free Recall phase, participants are asked to recall and say as many words as they can that they have seen before. In the Immediate Recognition phase, 36 words are presented sequentially, including 18 target words and 18 distractor words related to the meaning of the target word; participants are asked to click on the words that they had previously seen. In the Delayed Recognition phase, after a 20-minute delay, 36 words are presented sequentially, including 18 target words and 18 distractor words (differing from those used in the Immediate Recognition phase) related to the meaning of the target word; participants are asked to click on the words that they had previously seen.

Rapid Visual Information Processing (RVP): You are asked to respond to target sequences of digits (e.g., 3-5-7, 2-4-6, 4-6-8) as soon as possible by clicking a button at the centre of the device screen. The test is delivered in two parts: a 2-min practice stage that is not scored and a 3-min test stage. The level of difficulty varies with either 1- or 3-target sequences that the participant must watch for simultaneously.

Stockings of Cambridge (SOC): Two groups of patterns containing three colored balls were displayed on the computer screen in a specific configuration. The participants needed to move the balls on the bottom of screen to match with the goal set on the top using as few moves as possible. The number of moves increased from 2 to 4. In the motor control phases inserted in the test, the software waited for 5 s and then moved a ball in the example configuration, and the subject needed to follow what it did. The duration after each problem was 3 s.

###### What do you need to note?

1. If you cannot come to the hospital on the day of your visit, please contact us earily.
2. Please contact us before you change the medication you are taking or other brain stimulation treatment you are currently receiving from doctors.
3. tDCS is not available as a treatment for cognitive side effects of ECT because it is not approved.

###### The risk and burdens

According to a systematic review, serious adverse events is unlikely to occur, and common adverse events include tingling sensations, itching, mild transient redness of the skin and discomfort in the region of stimulation, moderate fatigue, difficulty concentrating, and headache. In case of serious adverse effects, appropriate medical and nursing care will be provided until the symptoms have ceased. We will not charge you anything. And fare for participation in the study will be reimbursed upon completion of the trial.

###### Benefits

We conducted a preliminary study of 30 patients with cognitive side effects who underwent tDCS (same as this one) while undergoing ECT course, cognitive function as assessed by the CANTAB showed that, there was a trend of improvement in active group compared to sham group. Thus, participation in this study may accelerate your recovery from the cognitive side effects. Otherwise, this study provides knowledge about whether the intervention really works.

###### Withdrawal of consent after participation in study

You can decide for yourself whether to participate or continue this study. You will not be disadvantaged in any way if you refuse to participate in this study. You can exit this trial at any time, and you will not be disadvantaged in any way, even if you have have started the study.

###### If you have any questions about this research

If you or your family have any questions or concerns about this study, please do not hesitate to contact us and call phone number is 18368536092. However, we may not be able to respond to you or answer your questions when it involves the privacy of other participants or intellectual property.

###### The system of this study

The name of the medical institution: Depart ment of Psychiatry, the First Affiliated Hospital of Chongqing Medical University.

The name of the principal investigator: Qinghua Luo, MD

Signing of Informed Consent Form

I have been fully briefed and understand the following regarding the "The ability of transcranial direct current stimulation to ameliorate cognitive side effects of electroconvulsive therapy in patients with depressive disorder: a randomized controlled trial", using a written explanation. I am participating in this study of my own free will.

**Participants**

Signature:

Date of Agreement:

**The proxy or caregiver**

Date of Agreement:

Signature:

After I introduced this trial to the patient in strict accordance with the informed consent form, the patient and proxy/caregiver agreed to participate in this trial.

Date of explanation:

Signature of Physician:
